# Синергия в действии ГАМК и гипогликемических препаратов

**DOI:** 10.14341/probl13257

**Published:** 2023-08-30

**Authors:** И. Н. Тюренков, Т. И. Файбисович, Д. А. Бакулин

**Affiliations:** Волгоградский государственный медицинский университет; Военно-медицинская академия имени С.М. Кирова; Волгоградский государственный медицинский университет

**Keywords:** ГАМК, β-клетки, α-клетки, сахарный диабет

## Abstract

Сахарный диабет (СД) является ведущей причиной преждевременной смертности и инвалидизации. Несмотря на значительное число препаратов, эффективность терапии, направленной на нормализацию уровня гликемии и предупреждение осложнений, не в полной мере удовлетворяет врачей и пациентов. Поэтому продолжается поиск новых подходов для профилактики и лечения СД и его осложнений. Значительные ресурсы используются для разработки новых препаратов, но в последнее время обосновывается возможность использования «старых» широкодоступных средств с вновь открытыми плейотропными свойствами. К таким могут быть отнесены препараты гамма-аминомасляной кислоты (ГАМК) и средства, прямо или косвенно активирующие ГАМК-ергическую передачу, обладающие выраженным панкреопротективным действием, что широко обсуждается в зарубежной литературе в последние 10–15 лет. Однако в отечественной литературе подобных публикаций немного.Установлено, что содержание ГАМК в β-клетках у пациентов с СД 1 и 2 типа снижается, и это коррелирует с тяжестью течения заболевания. Генетическая супрессия ГАМКА рецепторов вызывает значительное снижение массы β-клеток и стимулированной глюкозой секреции инсулина, что подтверждает важное значение ГАМК в обеспечении гомеостаза глюкозы и целесообразность восполнения дефицита ГАМК при СД ее дополнительным введением. Установлено, что у животных с СД ГАМК подавляет апоптоз и стимулирует регенерацию β-клеток, увеличивает β-клеточную массу и продукцию инсулина.Получены экспериментальные данные, свидетельствующие о синергетическом действии ГАМК при комбинированном применении с агонистами рецепторов глюкагоноподобного пептида-1 (ГПП-1), ингибиторами дипептидилпептидазы 4-го типа (ДПП-4) и ингибиторами натрий-глюкозного котранспортера 2 (НГЛТ-2), когда наблюдается более выраженное панкреопротективное действие, обусловленное снижением окислительного и нитрозативного стресса, воспаления, повышением уровня белка Клото, активности Nrf-2 и ферментов антиоксидантной защиты, подавлением активности NF-kB и экспрессии провоспалительных цитокинов. В итоге все это приводит к снижению апоптоза и гибели β-клеток, увеличению β-клеточной массы, продукции инсулина и одновременно снижению уровня глюкагона, инсулинорезистентности.В обзоре обосновывается целесообразность применения ГАМК и препаратов с положительным ГАМК-ергическим действием в комбинации с антидиабетическими средствами нового поколения: агонистами рецепторов ГПП-1, ингибиторами ДПП-4 и ингибиторами НГЛТ-2 с целью повышения их антидиабетического потенциала. Поиск осуществлялся в базах Pubmed, eLibrary, Medline по ключевым словам: сахарный диабет, гамма-аминомасляная кислота, глюкагоноподобный пептид-1, агонисты рецепторов ГПП-1, глюкозозависимый инсулинотропный пептид, ингибиторы дипептидилпептидазы, ингибиторы натрий-глюкозного котранспортера 2 типа. Поиск осуществлялся за период с 2000 по 2022 гг., но в обзоре представлены результаты исследований, опубликованные преимущественно за последние 3 года, что обусловлено требованиями журнала к максимальному объему работы и числу источников.

## Abstract (en)

Diabetes mellitus (DM) is the leading cause of premature death and disability. Despite a significant number of drugs, the effectiveness of therapy aimed at normalizing the level of glycemia and preventing complications does not fully satisfy doctors and patients. Therefore, the search for new approaches for the prevention and treatment of DM and its complications continues. Significant resources are used to develop new drugs, but recently the possibility of using «old» widely available drugs with newly discovered pleiotropic properties has been substantiated. These may include preparations of gammaaminobutyric acid (GABA) and agents that directly or indirectly activate GABAergic transmission, which have a pronounced pancreatic protective effect, which has been widely discussed in foreign literature over the past 10–15 years. However, there are few such publications in the domestic literature.It has been established that the content of GABA in β-cells in patients with type 1 and type 2 diabetes is reduced and this correlates with the severity of the disease. Genetic suppression of GABA receptors causes a significant decrease in the mass of β-cells and glucose-stimulated insulin secretion, which confirms the importance of GABA in ensuring glucose homeostasis and the advisability of replenishing the GABA deficiency in DM with its additional administration. It has been established that in animals with DM, GABA suppresses apoptosis and stimulates the regeneration of β-cells, increases β-cell mass and insulin production.Experimental data have been obtained indicating a synergistic effect of GABA when combined with glucagon-like peptide-1 (GLP-1) receptor agonists, DPP-4 inhibitors and sodium-glucose cotransporter 2 (SGLT-2) inhibitors, when a more pronounced pancreoprotective effect is observed, due to decrease in oxidative and nitrosative stress, inflammation, increase in the level of Klotho protein, Nrf-2 activity and antioxidant defense enzymes, suppression of NF-kB activity and expression of pro-inflammatory cytokines. As a result, all this leads to a decrease in apoptosis and death of β-cells, an increase in β-cell mass, insulin production and, at the same time, a decrease in glucagon levels and insulin resistance.The review substantiates the feasibility of using GABA and drugs with a positive GABAeric effect in combination with new generation antidiabetic agents: GLP-1 receptor agonists, DPP-4 inhibitors and SGLT-2 inhibitors in order to increase their antidiabetic potential.The search was carried out in the databases Pubmed, eLibrary, Medline. Keywords: diabetes mellitus, gamma-aminobutyric acid, glucagon-like peptide-1, GLP-1 receptor agonists, glucose-dependent insulinotropic peptide, dipeptidyl peptidase inhibitors, sodium-glucose cotransporter 2 inhibitors. The search was carried out from 2000 to 2022, but the review presents the results studies published mainly in the last 3 years, due to the requirements of the journal for the maximum amount of work and the number of sources.

## ВВЕДЕНИЕ

Сахарный диабет (СД) ввиду его широкой распространенности и тяжести осложнений является важнейшей медицинской, социальной и экономической проблемой [1]. В РФ число пациентов с СД за последние 20 лет увеличилось более чем в 2 раза: по данным регистра больных с СД, 1.01.2021 на диспансерном учете состояли 4 799 552 (3,23%) пациента [2]. По прогнозам Международной диабетической ассоциации, численность пациентов с СД к 2040 г. достигнет 642 млн человек [2].

Применение современных препаратов позволило значительно улучшить качество жизни лиц, страдающих СД. Однако более чем у 2/3 пациентов не удается достичь целевых значений гликемии, также не всегда удается предупредить потерю β-клеток поджелудочной железы и многочисленные осложнения СД. Это побуждает искать новые мишени и новые препараты, использовать комбинированную терапию, разрабатывать новые стратегии и новые технологии лечения СД [1][3][4].

СД 1 и 2 типа связан с прогрессирующей гибелью β-клеток при участии иммунной системы и воспаления, окислительного и нитрозативного стресса, глюкозо- и липотоксичности. Одним из направлений лечения СД является применение лекарственных средств, подавляющих апоптоз и гибель β-клеток и активирующих их пролиферацию и регенерацию, повышение продукции инсулина [5–9]. Такие свойства были отмечены у инкретинов, гамма-аминомасляной кислоты (ГАМК), ингибиторами натрий-глюкозного котранспортера 2 типа (НГЛТ-2) и др., но при монотерапии не всегда удается достичь нормогликемии, снижения апоптоза и гибели β-клеток, усиления их регенерации и увеличения β-клеточной массы, предупредить развитие осложнений СД [9].

С целью повышения эффективности антидиабетической терапии СД широко используется комбинация 2 и даже 3 гипогликемических препаратов, дающих синергетический эффект [4][10].

В последние годы опубликован ряд исследований, посвященных синергическому действию ГАМК и некоторых гипогликемических средств последнего поколения, отражающих их влияние на гомеостаз глюкозы, функционирование β-клеток, формирование дополнительных положительных терапевтических эффектов [9].

В данном обзоре мы приводим данные, свидетельствующие о синергическом взаимодействии ГАМК и препаратов с ГАМК-ергическим действием в комбинации с препаратами, оказывающими инкретиноподобное действие: агонистами рецепторов глюкагоноподобного пептида 1 (ГПП-1Р), ингибиторами дипептидилпептидазы 4 (ДПП-4) и НГЛТ-2, предварительно рассмотрев их класс-специфические свойства, включая и плейотропные.

Поиск осуществлялся в базах Pubmed, eLibrary, Medline по ключевым словам: сахарный диабет, гамма-аминомасляная кислота, глюкагоноподобный пептид-1, агонисты ГПП-1Р, глюкозозависимый инсулинотропный пептид (ГИП), ингибиторы дипептидилпептидазы, ингибиторы натрий-глюкозного котранспортера 2. Поиск осуществлялся за период с 2000 по 2022 гг., но в обзоре представлены результаты исследований, опубликованные преимущественно за последние 3 года, что обусловлено требованиями журнала к максимальному объему работы и числу источников.

## РОЛЬ ГАМК В РЕГУЛЯЦИИ УГЛЕВОДНОГО ОБМЕНА

Со времени открытия ГАМК и ее тормозных свойств в головном мозге (ГМ) многое из наших представлений о ее физиологической роли существенно изменилось. Было установлено, что ГАМК определяется практически во всех тканях и органах. В тканях поджелудочной железы содержание ГАМК сопоставимо с таковым в ГМ и высоко экспрессируется в α- и β-клетках островков Лангерганса [6][11]. Содержание ГАМК в β-клетках у пациентов с СД 1 и 2 типа снижается, и нарушается секреция инсулина [12][13]. Это подтверждает утверждение, что потеря ГАМК коррелирует с патогенезом СД [8].

В островках поджелудочной железы в инсулин-продуцирующих β-клетках экспрессируются ГАМКА- и ГАМКВ-рецепторы, которые кодируются разными внутриклеточными путями и формируют различные механизмы передачи сигналов [6][12][14][15]. После высвобождения ГАМК влияет на активность α- и β-клеток поджелудочной железы через ионотропные ГАМКА-рецепторы и метаботропные ГАМКB-рецепторы. Действие ГАМК на α- и β-клетки различается, на первые оказывает ингибирующее действие, снижая продукцию и высвобождение глюкагона, на β-клетки — стимулирующее действие, увеличивая секрецию инсулина [15].

ГАМК в β-клетках поджелудочной железы через ГАМКА-рецепторы активирует кальций-зависимые сигнальные пути и вольтаж-зависимые каналы, запускает сигнальные каскады PI3K/Akt, CREB-IRS-2 и SIRT-1, что может быть определяющим в регуляции массы и функций β-клеток [11][15].

Ожирение играет ключевую роль в развитии инсулинорезистентности, СД и его осложнений [16][17]. Поэтому снижение инсулинорезистентности является самостоятельной задачей профилактики и лечения СД. ГАМК обладает терапевтическим потенциалом против ожирения, что связано с подавлением окислительного стресса и стресса эндоплазматического ретикулума (ЭР). ГАМК снижала экспрессию адипогенного фактора транскрипции PPAR-y в жировой ткани и печени мышей, получавших богатую жиром и высококалорийную пищу. С помощью ГАМК контролируется активность НАДФН-оксидазы (NOX4) при дисметаболизме, связанном с высокожировой и богатой углеводами пищей, вызывающий распад IRE-1a, сульфонировавший RIDD-SIRT. Введение ГАМК значительно повышало уровни AMPK и SIRT-1 у мышей с ожирением [17], снижало инсулинорезистентность, увеличивало уровень гликогена в печени, улучшало липидный спектр, снижало уровень глюкозы и гликированного гемоглобина [18][19]. ГАМК значительно снижала инсулинорезистентность за счет активации экспрессии гена переносчика глюкозы (GLUT-4), подавления пути глюконеогенеза в печени, снижения экспрессии генов FOXO1 и Pepck и одновременного увеличения экспрессии генов IRS-2 и Akt2 [18]. ГАМК стимулирует фосфорилирование CREB через цАМФ-элемент связывающий белок (AMPK), известный как ключевой фактор транскрипции, ответственный за поддержание гена инсулина и выживание β-клеток, что способствует увеличению β-клеточной массы. Трофические эффекты ГАМК формируются через активацию сигнальных путей Akt и CREB независимо друг от друга, но для оптимального ответа необходима активация обоих путей. Участие Ca2+ и CREB и зависимая от них активация глюкозой IRS-2 β-клеток важна для пластичности ответа β-клеток на повышенную потребность в инсулине. ГАМК-индуцированная активация CREB одновременно связана с повышенным уровнем экспрессии транскрипта IRS-2 [6][15][20].

Роль ГАМК-ергической системы в регуляции углеводного обмена подтверждается и тем, что генетическая супрессия ГАМКA-рецепторов значительно снижала массу β-клеток и стимулированную глюкозой секрецию инсулина, что приводило к нарушению гомеостаза глюкозы [8][9].

СД 1 и 2 типа сопряжены с потерей β-клеток и β-клеточной массы и, вследствие этого с недостаточной продукцией инсулина и гипергликемией [5][6][9][12]. Данные последних лет свидетельствуют о выраженном митогенном и панкреопротективном действии ГАМК и веществ с ГАМК-ергическим действием. Мишени, через которые ГАМК оказывает это действие (белок Клото, NF-kB, Nrf2, SIRT, FOXO, PI3K/Akt, CREB-IRS2 и др.), объясняют способность ГАМК подавлять апоптоз, гибель, дедифференцировку, а с другой стороны — активировать репликацию, регенерацию, дифференцировку β-клеток, увеличение β-клеточной массы и повышение секреции инсулина [8][9][15][20].

Важным свойством ГАМК у животных с СД является повышение продукции белка Клото (БК), который подавляет активность транскрипционного ядерного фактора NF-kB, повышает экспрессию Nrf-2 и вследствие этого повышает активность ферментов антиоксидантной системы, снижает продукцию активных форм кислорода, провоспалительных цитокинов, экспрессию индуцибельной NO-синтазы [14][21–23].

Важными для лечения СД 1 и 2 типа являются иммуномодулирующие свойства веществ с ГАМК-позитивным действием. Эти свойства подтверждены и в клинических исследованиях, в которых установлено, что ГАМК может снижать провоспалительные профили мононуклеарных клеток периферической крови и СД4+-Т-клеток у пациентов с СД1 типа [14][23].

Изучение механизмов участия системы ГАМК в регуляции функции, чувствительности β-клеток к глюкозе, углеводного гомеостаза открывает новые аспекты терапевтического антидиабетического потенциала ГАМК и веществ, активирующих ГАМКА- и ГАМКВ -рецепторы [6][9][12][15][20][24].

Таким образом, защитная функция ГАМК в клетках поджелудочной железы, в других процессах регуляции углеводного обмена многогранна, не сводится к одному фактору или сигнальному пути и не полностью изучена.

Все представленное может лежать в основе синергетического действия при комбинации ГАМК с разными антидиабетическими средствами.

## СИСТЕМА ИНКРЕТИНОВ В РЕГУЛЯЦИИ УГЛЕВОДНОГО ОБМЕНА

СД 2 типа сопряжен с уменьшением выработки инкретинов и/или повышенной их деградацией ферментом (ДПП-4), что способствует повышению уровня глюкозы, так как синтез и высвобождение инсулина на 60–70% зависят от участия инкретинов [1].

В последние годы значительное место в лечении СД 2 типа заняли инкретиномиметики: агонисты ГПП-1Р, коагонисты рецепторов ГПП-1 и ГИП и ингибиторы ДПП-4 [1][2][16].

Глюкагоноподобный пептид-1 (ГПП-1) кодируется проглюкагоном, белком предшественником из 158 аминокислот, который экспрессируется в кишечнике, поджелудочной железе и нейронах различных областей ГМ [16]. В ГМ и кишечнике проглюкагон под действием прогормонконвертазы 1/3 (PC1/3) расщепляется на ГПП-1, ГПП-2, глицентин, родственный глицентину полипептид (GPP) и оксинтомодулин. ГПП-1 выделяется в ответ на прием пищи (жиров, углеводов и белков) в двух эквивалентных формах: ГПП-1(7–37) и ГПП-1(7–36NH2) и еще меньше в виде ГПП-1(1–37) или ГПП-1(1-36NH2) [1][25]. ГПП-1 стимулирует секрецию инсулина, ГАМК, соматостатина, который ингибирует секрецию глюкагона, что предупреждает выраженное постпрандиальное повышение гликемии.

ГИП образуется в результате расщепления pro-ГИП из 153 аминокислот под действием PC1/3 в энтероэндокринных К-клетках верхнего отдела кишечника и α-клетках поджелудочной железы. Большая часть циркулирующего ГИП относится к ГИП(1–42) и более короткой форме ГИП(1–30NH2). ГИП секретируется в большей мере в ответ на прием жира. ГПП-1 и ГИП взаимодействуют со своими рецепторами, принадлежащими к семейству рецепторов глюкагона.

ГИП-рецепторы экспрессируются в эндокринной поджелудочной железе с сопоставимыми количествами микро-РНК в α-, β-, и δ-клетках, адипоцитах, эндотелии сердца и сосудов, различных областях ГМ (гипоталамусе, гиппокампе, коре и др.) [16][26]. После приема пищи, через 10–15 мин продукция ГПП-1 может возрастать с 5 до 40 пмоль/л, ГИП — с 20–30 до 300 пмоль/л [1][16]. Стимуляция секреции инсулина под влиянием ГПП-1 и ГИП происходит только при высоких уровнях гликемии, как только уровень глюкозы приближается к норме (4,5 ммоль/л) инсулиноактивирующий эффект ГПП-1 исчезает [1][27][28].

Действие ГПП-1 и ГИП кратковременно (1–2 и 6 мин соответственно), так как они подвергаются быстрому расщеплению ферментом ДПП-4, поэтому блокада ДПП-4 приводит к выраженному повышению уровня инкретинов и инсулина, снижению уровней гликемии и гликированного гемоглобина [29][30].

До недавнего времени ГИП считался непривлекательной терапевтической мишенью для лечения СД 2 типа. В последние годы установлено, что снижение резистентности к ГИП может улучшить гликемический контроль [31]. Также было установлено, что ГИП, помимо гликемических эффектов, может замедлять резорбцию костей, регулировать липидный обмен [27]. Данные о роли ГИП в регуляции энергетического и липидного метаболизма противоречивы. Например, мыши с полной потерей ГИП-рецепторов имеют сниженную массу тела на фоне высокожировой диеты. Это побудило разработку антагонистов ГИП-рецепторов для лечения ожирения. Однако влияние таких препаратов на массу тела незначительное, а их комбинация с агонистом ГПП-1Р снижает массу тела больше, чем это возможно при монотерапии любым из них [16]. Для дальнейших исследований более интересными оказались агонисты рецепторов ГИП и двойные агонисты — к ГПП-1- и ГИП-рецепторам одновременно. Первый разработанный коагонист рецепторов ГИП и ГПП-1 — соединение MAR-709 — был снят на 3-й фазе клинических исследований. Судьба другого агониста рецепторов ГИП и ГПП-1 — препарата тирзепатид, разработанного Eli Lilly, более оптимистична. Тирзепатид, вводимый в виде подкожной еженедельной инъекции, вызывал значительное снижение уровня глюкозы, инсулинорезистентности, массы тела, корректировал дислипидемию. Крупномасштабное клиническое исследование подтвердило, что тирзепатид улучшал контроль гликемии, приводил к потере массы тела до 20% у взрослых с индексом массы тела 30 и более после 72-недельного наблюдения.

Очевидно, поиск агонистов рецепторов ГИП и ГПП-1 будет продолжен.

ДПП-4 — уникальная мембраносвязанная аминопептидаза с молекулярной массой 100 кДа, которая с высокой селективностью расщепляет N-конец пептидов, содержащих пролин или аланин в предпоследнем положении. ДПП-4 присутствует в растворимой форме в различных биологических жидкостях (плазме, спинномозговой, синовиальной, семенной жидкости, желчи), в связанной с мембраной форме в почках, слизистой оболочке кишечника, гепатоцитах и эндотелиоцитах [30]. Фермент ДПП-4 широко экспрессируется на Т- и В-клетках, естественных клетках-киллерах, макрофагах, гемопоэтических клетках-предшественниках, он также действует как модулятор пролиферации Т-клеток [30], что может определять его иммунотропное действие.

ДПП-4 является ключевым модулятором инкретиновой системы и каталитически инактивирует ГПП-1 и ГИП. Быстрая инактивация этих инкретиновых гормонов под влиянием ДПП-4 ведет к недостаточному высвобождению инсулина и способствует нарушению гликемического контроля, что является вторичным по отношению к снижению продукции инкретинов при СД2 и повышенной инактивации их ДПП-4. При СД2 уровень ДПП-4 на 30% выше, чем у здоровых пациентов и положительно коррелирует с гликированным гемоглобином, размером адипоцитов, воспалением в висцеральной жировой ткани [29][30].

Ингибиторы ДПП-4 могут не только влиять на углеводный гомеостаз, но и тормозить ускоренное старение (сердечно-сосудистой, нервной и иммунной системы и др.), проявлять ангиопротективное действие, улучшать ремоделирование сосудов и ускорять восстановление миокарда после инфаркта и заживление диабетических ран, снижать риск ампутации нижних конечностей, оказывать нефропротективное действие [16][30].

В результате действия ДПП-4 на ГПП-1 образуются ГПП-1(9–37) и преобладающая форма ГПП-1(9–36NH2), которая не оказывает типичных действий интактного ГПП-1 (влияние на секрецию инсулина, глюкагона и аппетит) [30]. Однако, возможно, образующиеся продукты под влиянием ДПП-4 могут оказывать полезное действие на сердечно-сосудистую систему, а если это так, то ингибирование ДПП-4 может нарушать их положительные эффекты на сердечно-сосудистую систему, но это требует дополнительных исследований [29].

Субстратами ДПП-4 могут быть не только инкретины, но и многочисленные эндокринные пептиды, нейропептиды и хемокины, субстанция Р и нейропептид Y (NPY), мозговой натрийуретический гормон (BNP), белок стромальных клеток (SDF-1B), которые содержат аланин или пролин в N-концевом стартовом сайте. Биологическое значение таких эффектов ДПП-4 практически не изучено, но исключать плейотропные эффекты, связанные с их действием, не следует.

## КЛАСС-СПЕЦИФИЧЕСКИЕ СВОЙСТВА АГОНИСТОВ РЕЦЕПТОРОВ ГПП-1 И ИНГИБИТОРОВ ДПП-4, ВЗАИМОДЕЙСТВИЕ ИХ С ГАМК

Агонисты ГПП-1Р (лираглутид, эксенатид, ликсисенатид, албиглутид, семаглутид) снижают апоптоз β-клеток, стимулируют их пролиферацию, способствуют дифференцировке клеток предшественников в β-клетки [8], способствуют неогенезу β-клеток из протоковых клеток [9], повышают продукцию инсулина. При связывании ГПП-1 с рецептором происходит стимуляция секреции инсулина через активацию цАМФ-зависимого сигнального пути и высвобождения β-аррестина [16], ГПП-1 также инициирует сигнальные пути роста и выживания β-клеток, включающие PI3K/Akt [16]. Эти эффекты агонисты рецепторов ГПП-1 оказывают через сигнальные пути АМPK/mTOR и PI3K/Akt [8]. Агонисты ГПП-1, не влияя на экспрессию активаторов клеточного цикла, могут подавлять экспрессию ингибиторов клеточного цикла (p16, p18 и p21), что может лежать в основе их панкреопротективного действия [10].

Агонисты ГПП-1Р оказывают много дополнительных положительных эффектов, обусловленных широким распространением ГПП-1Р в различных органах и тканях (сердце, сосуды, почки, ГМ). Агонисты ГПП-1Р снижают частоту развития сердечно-сосудистых осложнений СД [16][29][32].

Многочисленные эффекты инкретинов сопряжены с участием ГАМК-ергической системы.

В большом числе работ установлено, что ГАМК и ГПП-1 оказывают взаимоусиливающее антидиабетическое действие. Так, ГАМК кишечной нервной системы посредством активации ГАМКA- и ГАМКB-рецепторов активирует выработку и высвобождение ГПП-1 из L-клеток. Точно так же ГПП-1 повышает чувствительность ГАМКА-рецепторов в β-клетках, и через них усиливается влияние инкретина на выработку и высвобождение инсулина [8]. ГПП-1 активирует фермент глутаматдекарбоксилазу в поджелудочной железе, что приводит к повышению синтеза и концентрации ГАМК в β-клетках и, соответственно, повышению продукции инсулина.

Сочетанное применение ГАМК и эксендина-4 (агониста ГПП-1Р) блокирует индуцированный цитокинами апоптоз β-клеток и способствует повышению секреции инсулина в островках человека. In vitro комбинация ГАМК и агониста ГПП-1Р активировала пути Akt, экспрессию SIRT-1 и БК, при участии которых формируется цитопротекция β-клеток [33].

Эксендин-4 оказывает митогенное действие на пролиферацию β-клеток путем активации стимуляторов клеточного цикла (циклин А и Cdk1) и активирующих пролиферацию факторов транскрипции через аденилат-циклазный путь кальциневрина/NFAT [9].

ГАМК и ГПП-1 способствуют репликации β-клеток, усиливают преобразование α-клеток в β-клетки и подавляют иммунные реакции и апоптоз клеток, поэтому они имеют ценное значение для регенерации β-клеток и лечения СД [6][10][15][24][34][35].

ГАМК-ергические нейроны в вентральном предбоковом ядре конечной полоски (alBST) у крыс экспрессируют ГПП-1Р. Нокдаун экспрессии GLP1R в alBST продлевает реакцию гипоталамо-гипофизарно-надпочечниковой оси на острый стресс. Учитывая данные о том, что ГАМК-ергическая проекция от вентрального alBST служит для ограничения реакции индуцированной стрессом оси гипоталамус-гипофиз-надпочечники, можно предположить, что передача сигналов ГПП-1 приводит к активации ГАМК-ергических вентральных нейронов alBST, которые проецируются непосредственно в паравентрикулярное ядро гипоталамуса (PVN) [20][32][35][36]. Эти данные раскрывают механизм взаимодействия ГПП-1Р и ГАМК, посредством которых alBST осуществляет ингибирующий контроль над эндокринной осью гипоталамус-гипофиз-надпочечники.

По всему мозгу ГПП-1Р расположены в ГАМК-ергических нейронах ГМ, в том числе в ядре солитарного тракта (NTS). Активация этих рецепторов повышает высвобождение ГАМК, что может лежать в основе различных психоневрологических эффектов ГАМК и ГПП-1, в том числе и анорексигенного действия [32].

Ряд агонистов ГПП-1Р — лираглутид и семаглутид — зарегистрированы как средства для лечения ожирения, снижающие аппетит и массу тела [16][37]. Есть основания предполагать, что комбинированное применение агонистов ГПП-1Р и препаратов с ГАМК-ергическим действием может существенно усилить фармакологический потенциал их анорексигенного действия [7], но это требует экспериментального подтверждения.

## СИНЕРГЕТИЧЕСКОЕ ВЗАИМОДЕЙСТВИЕ ГАМК И ИНГИБИТОРОВ ДПП-4

Совместное использование ГАМК и ситаглиптина в эксперименте на мышах с диабетом, индуцированным стрептозотоцином, уменьшает апоптоз и повышает регенерацию имплантированных β-клеток человека, а также повышает уровень эндогенного ГПП-1 и инсулина, улучшает гомеостаз глюкозы более выраженно, чем их раздельное применение [8][10].

Liu W. и соавт. [7] при пересадке β-клеток человека мышам с СД определяли TUNEL-методом количество апоптотических клеток. У нелеченых животных их насчитывалось примерно 1,5%, при раздельном применении ГАМК и ситаглиптина их число снизилось до 0,51% и при их комбинированном применении — до 0,3%, т.е. защита β-клеток от апоптоза была наиболее выражена при комбинированном, чем при их раздельном применении.

Shao W. и соавт. [39] отмеченное ими аддитивное действие ГАМК и ситаглиптина на β-клетках связывают с однонаправленным их ингибирующим действием на экспрессию тиоредоксин-взаимодействующего белка (TxNIP). Нокдаун TxNIP повышал уровни GLP-1, Pdx1, Nkx6.1 и Mafa, что ассоциировалось с повышенным ответом на ГАМК и GLP-1 при стимуляции секреции инсулина. Таким образом TxNIP может рассматриваться как общая мишень для ГАМК и препаратов с инкретиноподобным действием [39].

Представленные данные свидетельствуют, что добавление агонистов ГАМК к препаратам инкретиноподобного действия может повысить эффективность лечения обоих типов СД [8].

## ИНГИБИТОРЫ НГЛТ-2 — НОВЫЙ КЛАСС АНТИДИАБЕТИЧЕСКИХ СРЕДСТВ

## Общая характеристика ингибиторов НГЛТ-2.

НГЛТ представляют собой транспортеры натрия и глюкозы в почечных канальцах, кишечнике с низким сродством и высокой пропускной способностью, расположенные в проксимальных канальцах почек. НГЛТ обнаруживаются также в ГМ, печени, сердце, скелетных мышцах, щитовидной железе [40]. Эти переносчики в итоге обеспечивают 90% реабсорбции глюкозы в почках и кишечнике. Ингибиторы НГЛТ-2 связываются с НГЛТ-2 в люминальной мембране ранних сегментов (S1 и S2) нефрона, где они блокируют до 50% реабсорбции глюкозы. Физиологический почечный порог реабсорбции глюкозы соответствует гликемии 10 ммоль/л, но у пациентов с СД 1 и 2 типа почечный порог повышен. Ингибиторы НГЛТ-2, подавляя реабсорбцию глюкозы и увеличивая глюкозурию, приводят к потере 75 г глюкозы в день, что соответствует примерно 300 ккал/день, и стимулируют осмотический диурез (400 мл/день). Такое действие вызывает снижение гликемии и способствует снижению массы тела [40].

Установлено, что ингибиторы НГЛТ-2 — эмпаглифлозин, дапаглифлозин и др., снижая глюкозотоксичность, улучшают функцию β-клеток. Ингибиторы НГЛТ-2 уменьшают апоптоз и гибель β-клеток, увеличивают пролиферацию и массу β-клеток у мышей, подвергшихся их повреждению стрептозотоцином [41][42].

Ингибиторы НГЛТ-2, снижая глюкозотоксичность, оказывают антиоксидантное действие посредством активации АМФ-активированной протеинкиназы (АМРК), ингибируют экспрессию NOX4, iNOS в тканях крыс с СД и вследствие этого подавляют окислительный стресс, продукцию АФК, генерируемую NADH-оксидазами [21][43]. Ингибиторы НГЛТ-2 снижают продукцию провоспалительных цитокинов (IL-6, ФНО-а, МСР, хемерина), ослабляют воспалительные процессы в сосудах и сердечной мышце, улучшают сократительную функцию сердца, уменьшают фиброз сердца, ремоделирование миокарда, уменьшают эндотелиальную дисфункцию [44–46].

Кардиопротективные эффекты связывают с их действием на сердечный натрий-протонный обменник NHE1 [46]. Важно отметить, что кардиопротективный эффект НГЛТ-2 наблюдается и у больных без СД [3][46].

В настоящее время НГЛТ-2 представляют фармакотерапевтическую группу, важную для лечения диабета и профилактики его осложнений [3][46][47], а препараты, одновременно ингибирующие НГЛТ-2 и НГЛТ-1, такие как канаглифлозин и сотаглифлозин, остаются еще более интересными для клинического применения [3].

Одобренные в последние 10 лет в США и Европе пять высокоаффинных ингибиторов НГЛТ-2: дапа-, кана-, сота-, эрту- и эмпаглифлозин, имеющие наномолярное сродство к НГЛТ-2, получили широкое применение в лечении СД и среди гипогликемических средств отличаются специфическими свойствами.

## КЛАСС-СПЕЦИФИЧЕСКИЕ СВОЙСТВА ПРЕПАРАТОВ, ИНГИБИРУЮЩИХ НГЛТ-2

Препараты этой группы имеют высокий терапевтический потенциал, инсулиннезависимый механизм действия, эффективно снижают уровень глюкозы в крови и гликированного гемоглобина, массу тела, артериальное давление (АД), уровень мочевой кислоты [3]. Ингибиторов НГЛТ-2 от других гипогликемических препаратов отличает широкий спектр плейотропных свойств. Они проявляют противовоспалительное действие, снижая продукцию провоспалительных цитокинов, поляризацию макрофагов M2 [48], улучшают функцию эндотелия, оказывают антиатеросклеротическое действие, уменьшают сердечно-сосудистые [44][45] и почечные риски [49][50]. Ингибиторы НГЛТ-2 являются жирорастворимыми веществами и хорошо проникают в мозг, где взаимодействуют с НГЛТ и поэтому уменьшают неврологические осложнения [48][51]. Это определило их место в терапии СД 2 типа, с указанными сопутствующими патологиями [3][44]. Важными свойствами ингибиторов НГЛТ-2 является их способность улучшать липидный обмен [4], снижать инсулинорезистентность [52], массу тела [47][53]. Важно также отметить и высокий профиль безопасности ингибиторов НГЛТ-2.

## СИНЕРГИЯ В ПАНКРЕОПРОТЕКТИВНОМ ДЕЙСТВИИ ИНГИБИТОРОВ НГЛТ-2 И ГАМК

На модели СД 2 типа получены обнадеживающие результаты по сохранению β-клеточной массы и функции β-клеток при комбинированном применении ингибиторов НГЛТ-2 и ГАМК за счет снижения метаболической глюко- и липотоксичности. Эти препараты рассматриваются перспективными в совместных протоколах применения [41].

Эмпаглифлозин и ГАМК при совместном применении улучшали гомеостаз глюкозы, плотность островков и соотношение площади инсулинопродуцирующих клеток и площади островков, а также метаболизма липидов. При сочетанном применении ГАМК и ингибиторов НГЛТ-2 наблюдается снижение стресса ЭР и окислительного стресса в поджелудочной железе, ГМ, почках [43][51].

Установлено, что ингибиторы НГЛТ-2 и ГАМК повышают чувствительность к инсулину тканей мозга и сердца, что может быть обусловлено их стимуляцией АМРК, главного регулятора клеточной энергии [52] и это коррелирует с их антиоксидантным и противовоспалительным действием [19].

В последние годы в ряде обзоров в отечественных и зарубежных журналах подробно отражены современные представления о строении и функциях белка Клото (БК), его участии в регуляции различных функций в условиях нормы и патологий почек, поджелудочной железы, сердечно-сосудистой системы, нейродегенеративных и психических заболеваниях [33][54–56].

Сочетанное применение ГАМК и ингибиторов НГЛТ-2 приводит к повышению экспрессии БК при СД и к активации сигнальной системы TOR/AMPK/SIRT-1, подавлению апоптоза и гибели β-клеток, усилению их регенерации и повышению секреции инсулина. Это объясняет множественные положительные свойства ингибиторов НГЛТ-2: эндотелио-, ангио-, кардио-, нефро-, нейро- и энцефалопротективное действие [21][33][49][57]. Эмпаглифлозин значительно усиливал экспрессию и активность белка Клото у животных с почечным фиброзом, у которых снижена экспрессия БК. Нокаут БК значительно ослабляет панкреопротективное действие ГАМК и НГЛТ-2 [49].

Таким образом, ингибиторы НГЛТ-2 и ГАМК оказывают сходное действие на β-клетки поджелудочной железы (на многие сигнальные системы), активируя PI3K/Akt, AMPK, повышая уровень белка Клото и, опосредованно стимулируя Nrf-2 и ферменты антиоксидантной защиты, подавляя экспрессию Nf-KB, провоспалительных цитокинов и iNOS, что приводит к снижению выраженности воспаления, окислительного и нитрозативного стресса и вследствие этого апоптоза, и гибели β-клеток с увеличением β-клеточной массы и продукции инсулина [41][49][54].

## ЗАКЛЮЧЕНИЕ

Разработка инновационных препаратов требует значительных ресурсов. Альтернативой этому может служить выявление для уже известных препаратов новых мишеней, открывающих иные показания и перспективы для их применения в комплексной терапии.

К таким могут быть отнесены ГАМК и препараты с ГАМК-ергическим действием, которые судя по множеству публикаций способны улучшать функциональное состояние β-клеток, снижать окислительный стресс и воспаление. В настоящее время накопились данные о синергетическом действии ГАМК при комбинированном применении с агонистами ГПП-1Р, ингибиторами ДПП-4 и ингибиторами НГЛТ-2 с усилением панкреопротективного действия, что обусловлено снижением окислительного стресса и воспаления, повышением уровня белка Клото, активности Nrf-2 и ферментов антиоксидантной защиты, подавлением активности NF-kB и экспрессии провоспалительных цитокинов. Это приводило к снижению гибели β-клеток, увеличению β-клеточной массы, продукции инсулина и одновременно снижению инсулинорезистентности.

Выявление синергии в действии ГАМК и гипогликемических препаратов открывает новые перспективы для улучшения клинических протоколов комплексного лечения СД2. Такие комбинации могут дать не только усиление гипогликемического действия, но и могут расширить спектр плейотропных эффектов, снизить стоимость лечения. Что актуально с учетом высокой цены у препаратов новых групп.

## ДОПОЛНИТЕЛЬНАЯ ИНФОРМАЦИЯ

Источники финансирования. Работа выполнена при финансовой поддержке Гранта РНФ от 19 апреля 2021 №21-15-00192 «Панкрео-, эндотелио- и нейропротективные свойства производных ГАМК».

Конфликт интересов. Авторы декларируют отсутствие явных и потенциальных конфликтов интересов, связанных с содержанием настоящей статьи

Участие авторов. Тюренков И.Н., Файбисович Т.Н. и Бакулин Д.А. внесли значимый вклад в проведение поисково-аналитической работы и подготовку статьи. Все авторы одобрили финальную версию статьи перед публикацией, выразили согласие нести ответственность за все аспекты работы, подразумевающую надлежащее изучение и решение вопросов, связанных с точностью или добросовестностью любой части работы.

Благодарности. Выражаем искреннюю благодарность Соколовой Ю.О., Юдиной Л.И. за техническую помощь в подготовке рукописи к публикации.
